# Traumatic occult pneumothorax and ipsilateral hydatid cyst: A case report

**DOI:** 10.1016/j.ijscr.2023.108647

**Published:** 2023-08-13

**Authors:** Mohammad Yosof Alhomsi, Mohammad Badr Almoshantaf, Saad Bashar Homsieh

**Affiliations:** a Department of Neurosurgery, Ibn Al-Nafees Hospital, Damascus, Syria; b Department of Plastic Surgery, Ibn Al-Nafees Hospital, Damascus, Syria

**Keywords:** Pneumothorax, Hydatid cyst, Trauma, Case report, Emergency

## Abstract

**Introduction:**

Traumatic pneumothorax is a common chest condition that can be caused by a chest trauma. Hydatid cysts are also common, especially in Syria, and is caused by Echinococcus granulosis infection.

**Case presentation:**

We report a case of mutual presentation of pneumothorax and a large Hydatid cyst on the same chest side in an 18 years-old patient who got stabbed in the chest. The chest x-ray reveled well-defined, homogeneous radio-opacity lesion that is consistent with Hydatid cyst but no pneumothorax was observed. Later, the chest CT showed a small pneumothorax that coexist with the Hydatid cyst. The case was treated conservatively and the patient survived.

**Discussion:**

Some studies support treating asymptomatic trauma patients with occult PT with observation and placing a chest tube if still asymptomatic. Our case questions the management protocol for such a rare encounter as the stability status of the patient was poor, and there was a large hydatid cyst close to the chest wall.

**Conclusion:**

Physicians should be aware of the possible management solutions when dealing with similar cases, especially in emergency settings. Until clear guidelines are published for this matter, we recommend that high-level observation of the patient's vitals are the determining factor for suitable intervention.

## Highlights

• Mutual presentation of Traumatic pneumothorax and Hydatid cysts is rare in the literature.
• No current guidelines are available for the management of this coincidence.
• Both conservative and interventional management are discussed but require continues monitoring.

## 1 Introduction

Pneumothorax (PT) is a common complication of chest trauma. It is defined as the presence of air in the pleural space. Thoracic trauma is categorized into blunt or penetrating trauma, and the latter is less common but associated with a higher mortality rate [1]. The most common causes of penetrating chest trauma are gunshots, stabbings, and fragments from military explosive devices. Penetrating trauma can cause pneumothorax, pulmonary contusion, hemothorax, pulmonary laceration, pericardial effusion, or tamponade [2]. PT in penetrating chest trauma occurs because of transpleural injures that permit gas to accumulate between the parietal and visceral pleura, causing the lung to collapse partially or fully. Occult PT is one that is not visible on a plain CXR, but it is detected with thoracic computed tomography (CT) [3].

Infection with Echinococcus granulosis is the cause of hydatid cysts (HC). HC is most commonly found in the liver and lungs, although it can occur in any human body organ. Many hydatid cysts of the lung remain asymptomatic, especially in children and adolescents. They can reach large sizes due to a weakened immune response and the high elasticity of lung parenchyma [4,5]. The most common symptoms of pulmonary cystic echinococcosis include cough, chest pain, dyspnea, and hemoptysis. Many cases, just like ours, remain asymptomatic and are diagnosed incidentally. The significant complication is cyst rupture, which can cause acute hypersensitivity reactions, including anaphylactic shock [6]. The literature holds very few case reports of pneumothorax resulting from force trauma caused by rupture of a lung hydatid cyst in the pleural cavity. In our case, a patient developed a traumatic occult pneumothorax after suffering from a stab wound. After the chest X-ray, we accidentally discovered an undiagnosed, asymptomatic large hydatid cyst close to the stab wound. This case was written using the SCARE checklist criteria [7]. This case highlights the importance of a proper guideline to exist for similar cases. Also, it gives physicians a management plan if they face such an encounter.

## 2 Case presentation

**Fig. 1:**
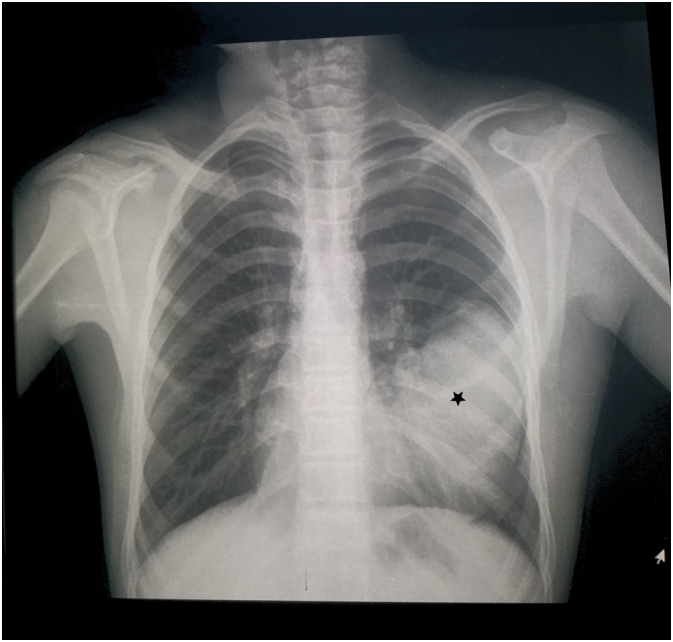
Chest X-ray on admission showing a large well-defined homogeneous radio-opacity (Black star).

**Fig. 2:**
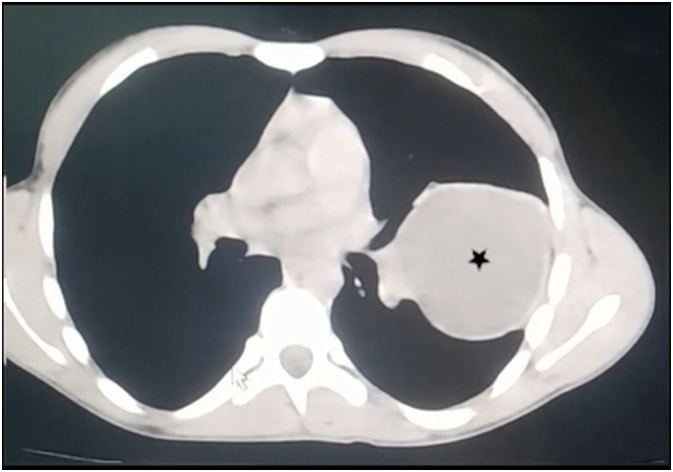
Chest CT scan showing a hydatid cyst lesion with homogenous content and smooth hyperdense walls (Black star).

**Fig. 3:**
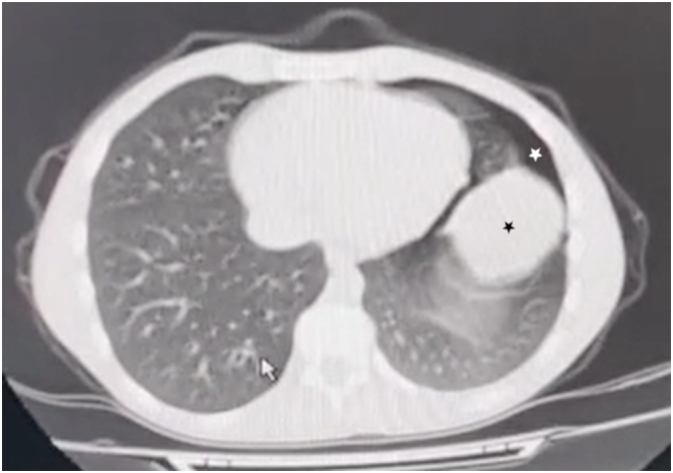
Chest CT scan showing a hydatid cyst lesion (Black start), and a pneumothorax (White star).

We present the case of an 18-year-old male who presented to the emergency department after a street fight. In attendance, he immediately stated that he was suffering from a stab wound in his left chest. He was responsive and alert, but in distress and agony. On examination, vital signs were stable and within normal limits. He presented with mild dyspnea. No cough or hemoptysis was noted. Lung auscultation revealed decreased breath sounds in the left thorax despite a normal pulse oximeter. An urgent chest X-ray was ordered and showed a large, well-defined, homogeneous radio-opacity with no evident pneumothorax (Fig. 1). HC was initially suspected because of its high prevalence rates in our region. Chest CT demonstrated a small left pneumothorax and a large well-circumscribed fluid attenuation lesion with homogenous content and smooth hyperdense walls (Fig. 2) (Fig. 3). The latter is radiologically consistent with an HC. No signs of rupture were observed. Laboratory findings were within normal limits. Physical examination revealed a stab wound in the left fifth intercostal space between the anterior and midaxillary lines with minimal bleeding. As such cases of stab wounds are at high risk of developing a one-way valve resulting in tension PT, close monitoring was advised. Also, we consistently observed the patient for any signs of a potential hypersensitivity reaction in the case of cyst rupture. After 48 h of active observation, the patient did not suffer from any abnormalities, and we noticed no signs of complications.

The patient was discharged with instructions to return in the event of worsening breathlessness. Surgical resection of the cyst was scheduled after six weeks of discharge and was done without any difficulties. Today, the patient is experiencing no relevant symptoms or complications and normally carries out daily activities.

## 3 Discussion

Management of PT varies by the cause, size, and degree of the symptoms and may involve observation, needle aspiration, and chest tube insertion [8]. The management of traumatic occult PT is controversial. Most studies support treating asymptomatic trauma patients with occult PT with observation and placing a chest tube if only the patient becomes symptomatic or if the patient requires positive-pressure ventilation [9]. In our case, the patient had a left traumatic occult pneumothorax after penetrating chest trauma with a large hydatid cyst in the middle and lower zones of the left chest. Because the PT was small, the stability status of the patient was poor, and there was a large hydatid cyst close to the chest wall, we decided to apply a preservative approach by observing and closely monitoring the patient. We reserved a tube thoracostomy in case of PT expansion or HC rupture.

Serial chest X-rays are usually indicated to detect delayed PT after a penetrating thoracic injury [10]. As we had already diagnosed the occult PT via CT, we used serial chest x-rays with a 3–4 h' intervals to detect possible progression of the PT or rupture of the HC. After 48 h of extensive observation, the patient was discharged. A combination of imaging and serology confirmed the HC diagnosis.

A chest tube should have been inserted under ultrasound guidance and with an expert hand to avoid accidental cyst injury if cyanosis or dyspnea had been observed during active observation. Surgical resection of the cyst after the monitoring period and before discharge was an option. The rationale behind such management was due to the large size of the cyst. But, in the end, we agreed on elective resection of the cyst 6 months post-discharge, and pathological analysis concluded that the cyst was indeed HC.

In a case by Shameem et al. [11], A PT has resulted from a spontaneously ruptured HC in non-traumatic settings. Due to the patient's unstable vitals, she was treated with an emergency intercostal chest tube and extensive resuscitation. Similar to Shameem et al., Kürkçüoğlu et al. present two cases of tension PT due to HC rupture, where both cases were subjected to surgical treatment after urgent tube thoracostomy [12]. We conclude that large HC alone is alarming enough to cause PT due to a spontaneous rupture, and while stabbing injury also forms a significant risk factor for developing traumatic PT, double caution is required when dealing with large HC and stab wounds, especially if on the same side.

## 4 Conclusion

Stabbing injuries can cause occult or normal pneumothorax. In asymptomatic cases, patients may be treated conservatively without chest tube insertion but must be monitored with serial chest X-rays to exclude any development. If an accidental unruptured hydatid cyst was detected ipsilaterally, it might be possible to continue active observation with additional alertness. A take-home message from this case is that despite the absence of clear guidelines to treat this case, the patient's vitals and sticking to the basics were the pillars of successfully managing a complicated case.

## Sources of funding

This research did not receive any specific grant from funding agencies in the public, commercial, or not-for-profit sectors.

## Ethical approval

This case report was reviewed and approved by ethics committee of the hospital in Sep/2021.

## Registration of research studies

Not applicable.

## Consent for publication

Written informed consent was obtained from the patient for publication of this case report and accompanying images. A copy of the written consent is available for review by the Editor-in-Chief of this journal on request.

## Author contribution

All authors have helped in writing and reviewing the manuscript.

## Declaration of competing interest

All authors declared no conflict of interest.
